# Identification of Four Oxidative Stress-Responsive MicroRNAs, miR-34a-5p, miR-1915-3p, miR-638, and miR-150-3p, in Hepatocellular Carcinoma

**DOI:** 10.1155/2017/5189138

**Published:** 2017-07-24

**Authors:** Yong Wan, Ruixia Cui, Jingxian Gu, Xing Zhang, Xiaohong Xiang, Chang Liu, Kai Qu, Ting Lin

**Affiliations:** ^1^Department of Geriatric Surgery, The First Affiliated Hospital of Xi'an Jiaotong University, Xi'an 710061, China; ^2^Department of Hepatobiliary Surgery, The First Affiliated Hospital of Xi'an Jiaotong University, Xi'an 710061, China; ^3^Department of Surgical Intensive Care Units, The First Affiliated Hospital of Xi'an Jiaotong University, Xi'an 710061, China

## Abstract

Increasing evidence suggests that oxidative stress plays an essential role during carcinogenesis. However, the underlying mechanism between oxidative stress and carcinogenesis remains unknown. Recently, microRNAs (miRNAs) are revealed to be involved in oxidative stress response and carcinogenesis. This study aims to identify miRNAs in hepatocellular carcinoma (HCC) cells which might involve in oxidative stress response. An integrated analysis of miRNA expression signature was performed by employing robust rank aggregation (RRA) method, and four miRNAs (miR-34a-5p, miR-1915-3p, miR-638, and miR-150-3p) were identified as the oxidative stress-responsive miRNAs. Pathway enrichment analysis suggested that these four miRNAs played an important role in antiapoptosis process. Our data also revealed miR-34a-5p and miR-1915-3p, but not miR-150-3p and miR-638, were regulated by p53 in HCC cell lines under oxidative stress. In addition, clinical investigation revealed that these four miRNAs might be involved in oxidative stress response by targeting oxidative stress-related genes in HCC tissues. Kaplan-Meier analysis showed that these four miRNAs were associated with patients' overall survival. In conclusion, we identified four oxidative stress-responsive miRNAs, which were regulated by p53-dependent (miR-34a-5p and miR-1915-3p) and p53-independent pathway (miR-150-3p and miR-638). These four miRNAs may offer new strategy for HCC diagnosis and prognosis.

## 1. Introduction

Hepatocellular carcinoma (HCC) is the fifth most common cancer and the third cause of cancer-related mortality worldwide [1]. It is established that hepatitis virus infection, as well as environmental carcinogens such as aflatoxin or chemical carcinogens, is associated with the development of HCC [2]. Although the carcinogenic mechanism of above risk factors varies, the common pathological process affected by those risk factors is hepatic chronic inflammation. Recruitment of inflammatory cells in hepatic environment and chemical mediator release, such as cytokines, chemokines, and reactive oxygen species (ROS), are considered to play a vital pathogenic role during hepatic carcinogenesis [3].

ROS are a group of chemically reactive molecules containing oxygen, which are mainly derived from cellular oxidative metabolism and play essential roles in the regulation of multiple cellular processes. During the development of many diseases, including malignant diseases, increasing ROS levels might lead to the imbalance of the pro-oxidant/antioxidant equilibrium and subsequently induce changes of intracellular molecules, including lipids, proteins, and nuclear acids [4]. Thus, the exploration of the intracellular molecules responsible for oxidative stress might enrich our understanding of molecular hepatic carcinogenesis.

Recently, accumulating evidences suggest that a series of small noncoding RNAs (microRNA) can be induced by oxidative stress. Several studies have examined the changes of the microRNA (miRNAs) expression profiles in varying cells upon treatment with hydrogen peroxide (H_2_O_2_) [5–14]. Unfortunately, inconsistent conclusion was made from those miRNome profiling studies. Confounding factors may include employment of different cell origins, various detection platforms, and application of different statistical methods.

To overcome those limitations, in the present study, we integrated these published relevant studies and performed a meta-analysis applying the rank aggregation method. We identified four common oxidative stress-responsive microRNAs in H_2_O_2_-treated cells. Moreover, we also evaluated the association between those oxidative stress-responsive microRNAs and p53, a key oxidative stress-responsive mediator in HCC cell lines. Finally, we validated the expression of identified miRNAs and their target genes in HCC tissues.

## 2. Materials and Methods

### 2.1. Literature Search and Inclusion Criteria

We performed a literature search in PubMed, Embase, and Web of Knowledge databases using search term combination of (miRNA^∗^ or microRNA^∗^ or mir-^∗^) and profil^∗^ and (oxidati^∗^ or hydrogen peroxide) and (cell^∗^ or cell line^∗^). We firstly screened all abstracts and selected potential abstracts for further full text evaluation. Only the original experimental studies that explored miRNA profile using a high-throughput miRNA expression profiling methods such as second-generation sequencing, polymerase chain reaction (PCR), or microarray-based high-throughput methods in H_2_O_2_-treated cells were included.

### 2.2. Data Extraction and Rank Aggregation Analysis

Rank lists of up- or downregulated miRNAs were extracted from the included studies. All included miRNA names were firstly standardized using miRBase (release 21.0). Rank aggregation method was implemented using an R package “Robust Rank Aggreg.” This method analyzed miRNAs that are ranked consistently better than expected and assigns a *P* value for each miRNA.

### 2.3. Pathway Enrichment Analysis

We firstly collected validated targets of each miRNA using miRTarBase database (http://mirtarbase.mbc.nctu.edu.tw/). Gene ontology (GO) biological process enrichment of those validated targets was performed using Database for Annotation, Visualization and Integrated Discovery (DAVID) v6.8 (https://david.ncifcrf.gov/). Fisher's exact test was used to identify the significant pathway terms. Heatmap was presented by log-transformed *P* value. Moreover, the combinatorial pathway enrichment analysis of multiple miRNAs was analyzed using “miRPath” algorithm (http://www.microrna.gr/miRPathv2).

### 2.4. miRNA-Gene Interaction Network Analysis

We firstly performed target prediction by using Targetscan website (http://www.targetscan.org/), and only cumulative-weighted context++ score< −0.4 was selected for further analysis. The interaction between miRNA and their predicted target networks was constructed using the Cytoscape software. In the validation group, the miRNA-gene interaction was presented based on their Pearson's correlation coefficient.

### 2.5. HCC Cell Line Culture

Four *TP53* wild-type HCC cell lines, including HepG2, SMMC-7721, HHCC, and SK-Hep-1, were selected for experimental validation. All cell lines were cultured using Dulbecco minimum essential medium (DMEM) supplemented with heat-inactivated 10% fetal bovine serum (FBS), 100 U/ml penicillin, and 100 *μ*g/ml streptomycin. Cells were incubated at 37°C and 5% CO_2_ in a HF100 chamber (Heal Force, Hong Kong, China).

### 2.6. Pro- and Antioxidant Treatment

For pro-oxidant treatment, HCC cell lines were firstly synchronized by serum starvation overnight and then treated with 200 *μ*M of H_2_O_2_ for 24 h. For antioxidant treatment, cells were preincubated with N-acetylcysteine (NAC, 5 mM) for 6 hours before exposure to H_2_O_2_. Following treatment, cells were harvested for further analysis.

### 2.7. Transfection of siRNA

RNA interference mediated by duplexes of 21-nucleotide RNA was performed in HCC cell lines. siRNA-*TP5*3 (5′-CUGGAAGACUCCAGUGGUA-3) and control-siRNA were synthesized by GenePharma (Shanghai, China). Transfection of siRNA (100 nM) was carried out with Lipofectamine 2000 (Invitrogen, Carlsbad, CA, USA) according to the manufacturer's protocols.

### 2.8. Quantitative Reverse Transcription-Polymerase Chain Reaction (qRT-PCR)

Total RNA was extracted from cultured cells using Trizol reagent (Invitrogen, Carlsbad, CA, USA). qRT-PCR was performed using the SYBR® PrimeScript™ miRNA RT-PCR Kit and SYBR Premix Ex Taq™ (TaKaRa Biotechnology, Dalian, China). All primers of selective miRNAs were also synthesized by TaKaRa. The miRNA expression was assayed in triplicate and normalized to U6. The relative miRNA expression was calculated using the comparative-Ct (△△Ct) method.

### 2.9. Public Datasets for Validation

To validate the expression of miRNAs and their targets in HCC tissues, we used public datasets containing 100 paired HCC tissue samples (GSE62007 and GSE62043). GSE62007 dataset was conducted on miRXplore Microarray platform, and GSE62043 dataset was conducted on Agilent-014850 Whole Human Genome Microarray platform. For hepatocellular carcinoma, The Cancer Genome Atlas (TCGA) dataset were used to explore the prognosis values of four miRNAs in HCC. The data were obtained from SurvMicro web tool (http://bioinformatica.mty.itesm.mx/SurvMicro).

### 2.10. Statistical Analysis

All data in this study were analyzed by SPSS 11.0 software (SPSS Inc., Chicago, IL, USA) and expressed as mean ± standard error of measurement (SEM). Analysis of continuous variables was performed using a Student *t*-test. Expression correlation between variables was tested by Pearson's correlation analysis. *P* < 0.05 was considered statistically significant.

## 3. Results

### 3.1. Identification of Oxidative Stress-Responsive miRNAs

**Figure 1:**
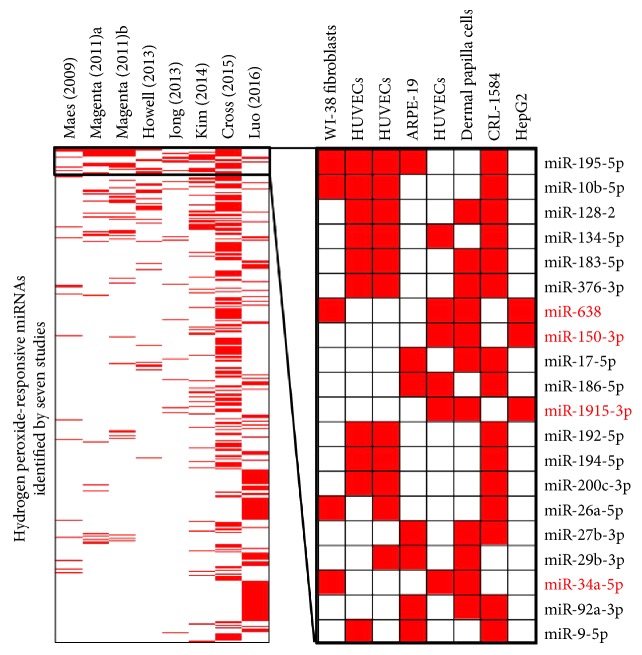
Heatmap of miRNAs reported by nine miRnome profiling studies. One selected miRNA was presented as red or white according to whether it was report by one study or not.

**Figure 2:**
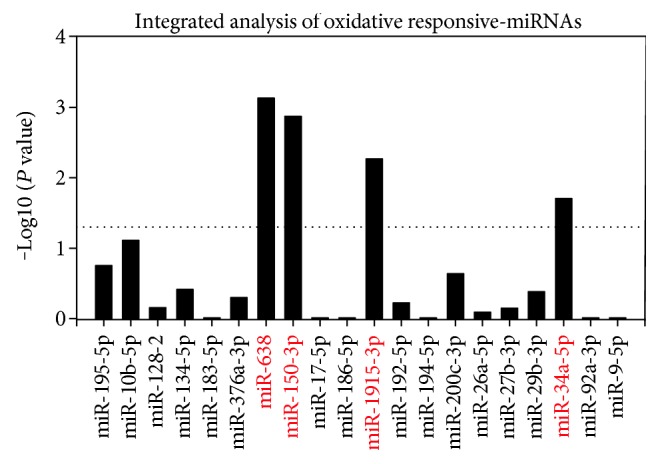
Integrated analysis of oxidative responsive-miRNAs by RRA method. The column was presented according to −Log_10_ (*P* value) of each miRNA which was calculated by RRA method.

A total of 11 studies from 10 published articles were screened [5–14], and 8 studies were based on human cells [6, 7, 10–14], which were selected for further analysis. In summary, 504 aberrantly expressed miRNAs were recorded, including 404 upregulated and 99 downregulated miRNAs. Among them, twenty upregulated miRNAs were reported by more than three studies (Figure 1). By applying a rank aggregation analysis, we identified four oxidative stress-responsive miRNAs which were significant upregulated under H_2_O_2_ treatment, including miR-638, miR-150-3p, miR-1915-3p, and miR-34a-5p (Figure 2).

### 3.2. Validation of Oxidative Stress-Responsive miRNAs in HCC Cell Lines

**Figure 3:**
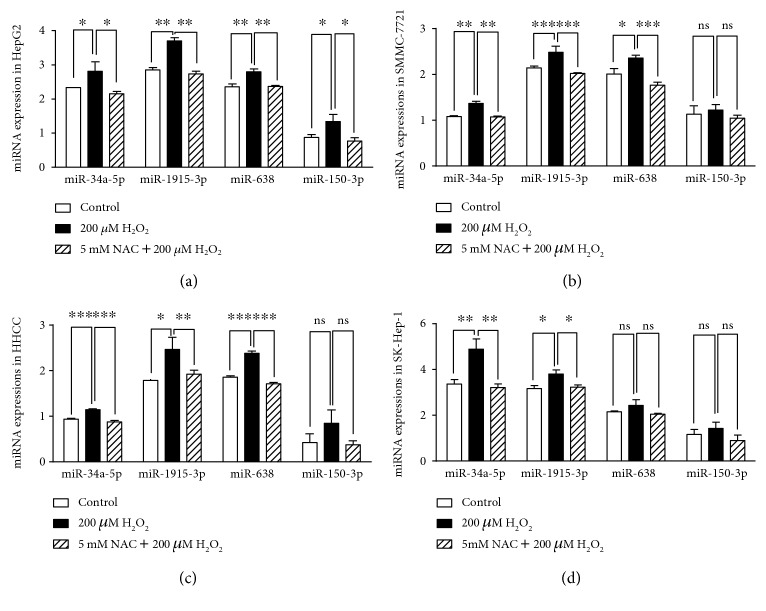
Expression of four oxidative stress-responsive miRNAs in different HCC cell lines. The expressions of miR-34a-5p, miR-1915-3p, miR-638, and miR-150-3p were measured by qRT-PCR method in HepG2 (a), SMMC-7721 (b), HHCC (c), and SK-Hep-1 (d). All HCC cell lines were treated with saline, 200 *μ*M H_2_O_2_ or 5 mM NAC + 200 *μ*M H_2_O_2_, respectively. All these experiments were conducted in triplicate. ^∗^*P* < 0.05; ^∗∗^*P* < 0.01; ^∗∗∗^*P* < 0.001; ns, no significant.

To further explore the expression changes of identified miRNAs in oxidative stress response in HCC, we treated four *TP53* wild-type HCC cell lines with 200 *μ*M H_2_O_2_ (the most widely used concertation) for 24 h and measured expression levels of four miRNAs (miR-34a-5p, miR-1915-3p, miR-638, and miR-150-3p). Our data revealed that the expressions of four miRNAs were all significantly increased after H_2_O_2_ treatment. By contrast, when preincubation with 5 mM NAC for 6 h, the upregulated expressions of miR-34a-5p, miR-1915-3p, miR-638, and miR-150-3p caused by H_2_O_2_ treatment were abrogated (Figure 3). The above results provide evidence that the expressions of miR-34a-5p, miR-1915-3p, miR-638, and miR-150-3p were tightly associated with intracellular oxidative stress status in HCC cell lines.

### 3.3. Function Prediction of Oxidative Stress-Responsive miRNAs

**Figure 4:**
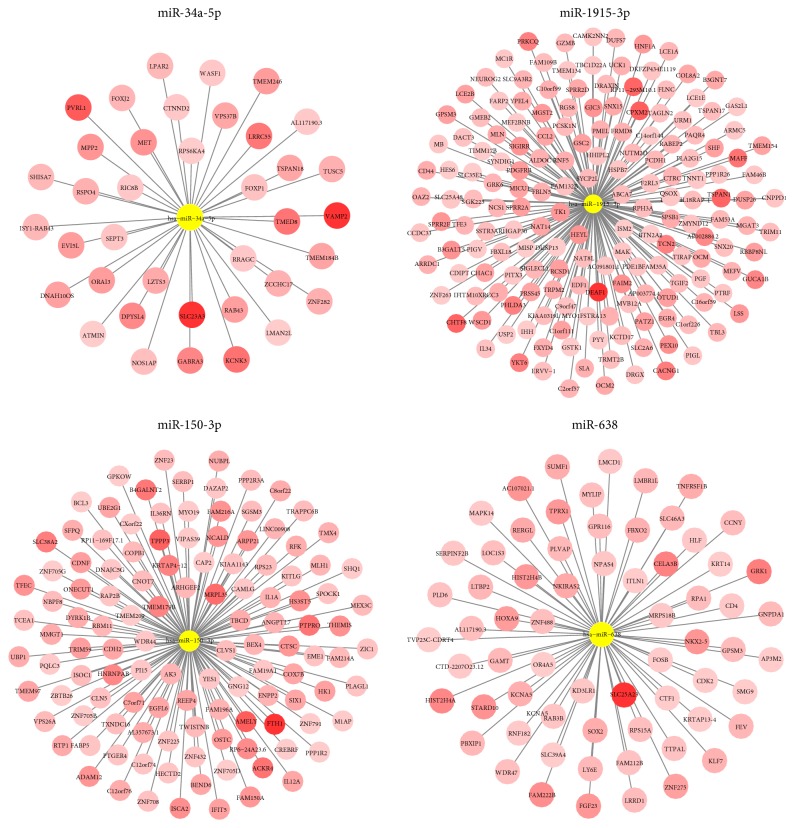
The interaction network between four miRNAs and their predicted targets.

**Figure 5:**
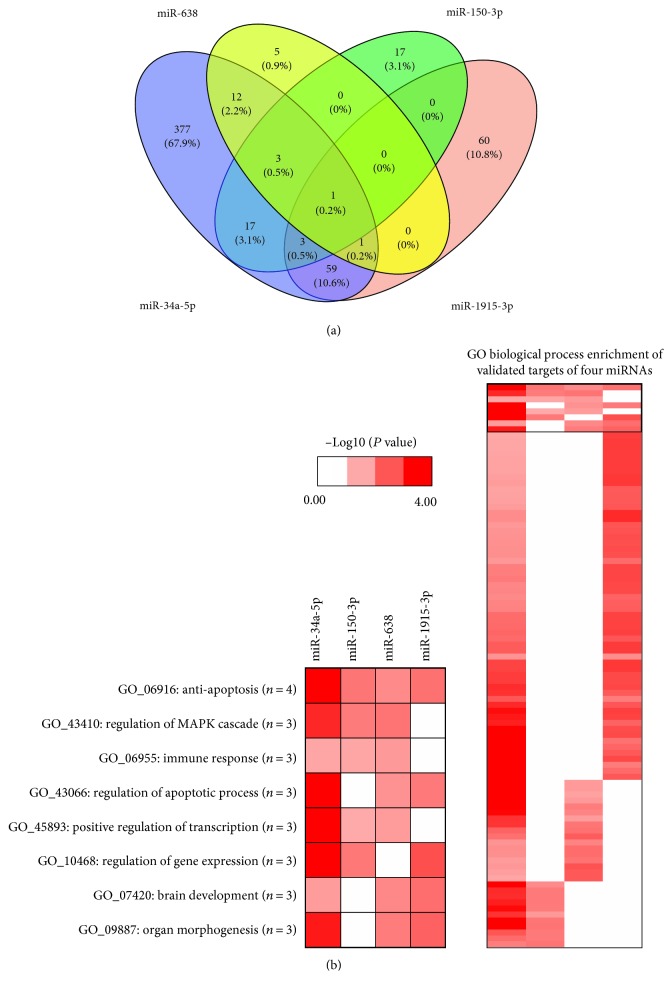
Pathway enrichment analysis of four oxidative stress-responsive miRNAs. (a) Venn diagram of pathways of four oxidative stress-responsive miRNAs. (b) Heatmap of pathways of four oxidative stress-responsive miRNAs. Range of colors (white to deep red) shows the −Log_10_ (*P* value of pathway enrichment analysis) (low to high).

We firstly performed target prediction by using Targetscan web tool, and the miRNA-target interaction was visualized in Figure 4. To further evaluate biological functions of four miRNAs, we selected their experimental validated targets for GO pathway enrichment analysis. The number of enriched GO biological process of each miRNA ranged from 22 to 474 (Figure 5(a)). By conducting a heatmap of *P* values derived from Fisher's exact test, four miRNAs were shown to be all closely related to antiapoptosis pathways (Figure 5(b)). Furthermore, combinatorial pathway enrichment analysis revealed that those four oxidative stress-responsive miRNAs were related to p53 signaling pathway (Table 1). Previous studies had clearly demonstrated the closely association between p53 signaling pathway and oxidative stress [15, 16], suggesting a critical role of four miRNAs (miR-34a-5p, miR-1915-3p, miR-638, and miR-150-3p) in oxidative stress response.

### 3.4. miR-34a-5p and miR-1915-3p Are Regulated by p53 under Oxidative Stress

**Figure 6:**
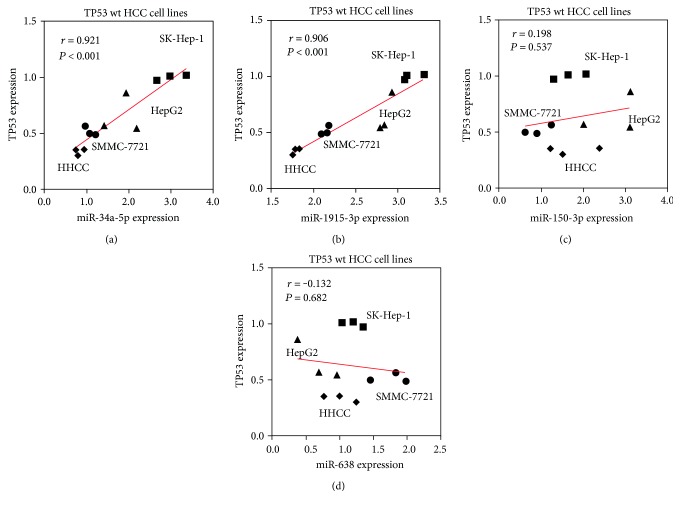
The expression correlation between four miRNAs and *TP53*. (a) miR-34a-5p, (b) miR-1915-3p, (c) miR-150-3p, and (d) miR-638.

**Figure 7:**
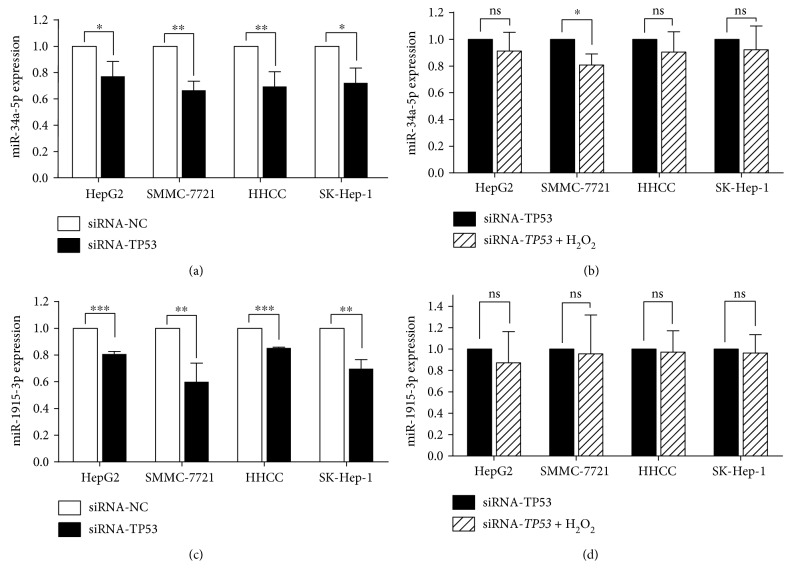
miR-1915-3p and miR-34a-5p are regulated by p53. (a, c) After transfection of siRNA-*TP53*, the expression of miR-34a-5p and miR-1915-3p was analyzed by qRT-PCR. (b, d) After transfection of siRNA-*TP53*, the expression of miR-34a-5p and miR-1915-3p was measured with or without H_2_O_2_ treatment. ^∗^*P* < 0.05; ^∗∗^*P* < 0.01; ^∗∗∗^*P* < 0.001; ns, no significant.

Since our data suggested an association between oxidative stress-responsive miRNAs and p53 signaling pathway, we wondered whether the expressions of these miRNAs in oxidative stress response are regulated by p53. Evidences from our [17, 18] and other groups [19] suggested that p53 was upregulated in response to oxidative stress in HCC. Thus, by employing four HCC cell line data, we quantified the correlation coefficient between p53 and four oxidative stress-responsive miRNAs expression. We found that p53 gene (*TP53*) was statistically positively correlated to the expressions of miR-34a-5p and miR-1915-3p (*P* < 0.05), but not miR-638 and miR-150-3p (*P* > 0.05) (Figure 6), indicating that p53 might regulate the miR-34a-5p and miR-1915-3p function in oxidative stress response. To further verify this hypothesis, we then examined the miR-34a-5p and miR-1915-3p expressions in siRNA-*TP53*-transfected HCC cells. As shown in Figures 7(a) and 7(c), the miR-34a-5p and miR-1915-3p expression levels were significantly decreased by knocking down of *TP53* (all *P* < 0.05). Furthermore, knocking down of *TP53* was shown to be able to attenuate the increased expression of miR-34a-5p and miR-1915-3p induced by H_2_O_2_ treatment, providing solid evidence for the regulatory roles of p53 in miR-34a-5p and miR-1915-3p in oxidative stress response (Figures 7(b) and 7(d)).

### 3.5. Clinical Values of Four Oxidative Stress-Responsive miRNAs in HCC

**Figure 8:**
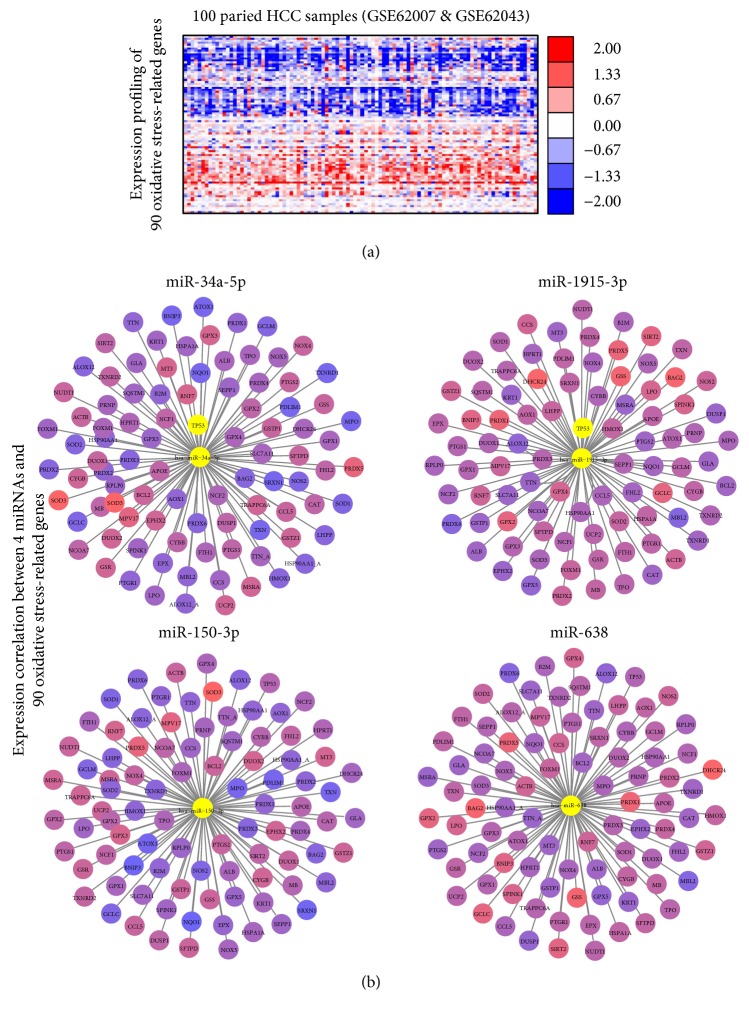
Four miRNAs and oxidative stress-related genes in HCC. (a) Heatmap of 90 oxidative stress-related gene expressions in HCC tissues with different miR-1915-3p status. (b) The expression correlation between four miRNAs and 90 oxidative stress-related genes. Range of colors (blue to red) was represented according to Pearson's correlation coefficient (low to high).

To gain insight into clinical values of four oxidative stress-responsive miRNAs in HCC, we firstly obtained 4 miRNAs and 90 oxidative stress-related gene profiling data based on 100 paired HCC samples (GSE62007 and GSE62043) from Gene Expression Omnibus (GEO, http://www.ncbi.nlm.nih.gov/geo/) (Figure 8(a)). By calculating the expression correlations between each miRNA and oxidative stress-related gene, we conducted a miRNA-gene interaction network (Figure 8(b)). Our data showed that miR-150-3p and miR-1915-3p were negatively correlated with more than half of oxidative stress-related genes (54/90 and 50/90, resp.). miR-34a-5p was negatively correlated with 44 out of 90 oxidative stress-related genes. Meanwhile, miR-638 was only negatively correlated with the least number of oxidative stress-related genes (38/90), which was partially consistent with previous study [20]. Moreover, tissue expression pattern also validated the positively correlation between p53 and miR-34a/miR-1915-3p with correlation coefficients of 0.13 and 0.16, respectively.

**Figure 9:**
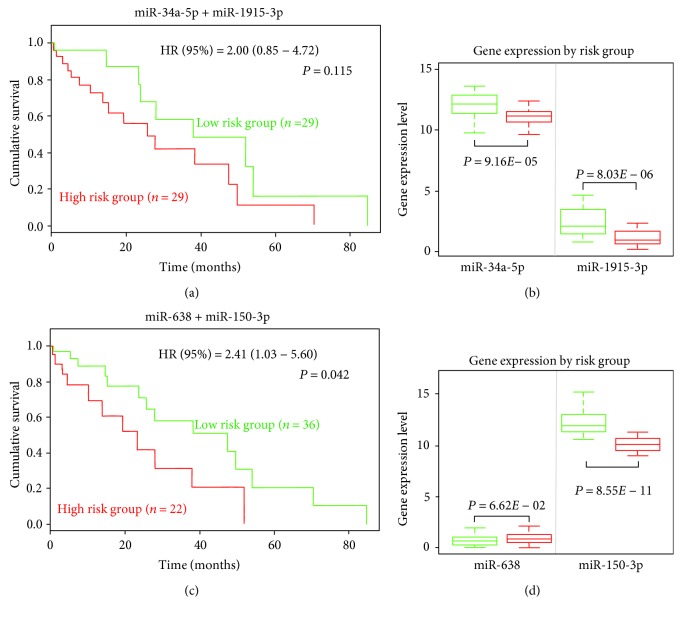
Prognosis values of four miRNAs in HCC. (a) Kaplan-Meier curve of overall survival for patients with different risk groups identified by miR-34a-5p and miR-1915-3p. (b) The expression levels of miR-34a-5p and miR-1915-3p in different risk groups. (c) Kaplan-Meier curve of overall survival for patients with different risk groups identified by miR-150-3p and miR-638. (d) The expression levels of miR-150-3p and miR-638 in different risk groups.

Next, we explored the prognosis values of four oxidative stress-responsive miRNAs in HCC patients. We obtained HCC TCGA data by using SurvMicro web tool (http://bioinformatica.mty.itesm.mx/SurvMicro) and conducted Kaplan-Meier analysis of HCC patients with different miRNA expression levels. We found that HCC patients with lower miR-34a and miR-1915-3p expression levels had shorter overall survival time, although we did not get a statistically significant *P* value which might due to a small sample size (Figures 9(a) and 9(b)). Similarly, we also found that miR-638 and miR-150-3p could predict HCC patients' survival time, with a *P* value of 0.042 (Figure 9(c)). It should be pointed out that the expression level of miR-638 in high-risk group was slightly higher than that in low risk group, with a nonsignificant *P* value (Figure 9(d)), which may warrant future larger study to confirm or refuse it.

## 4. Discussion

**Figure 10:**
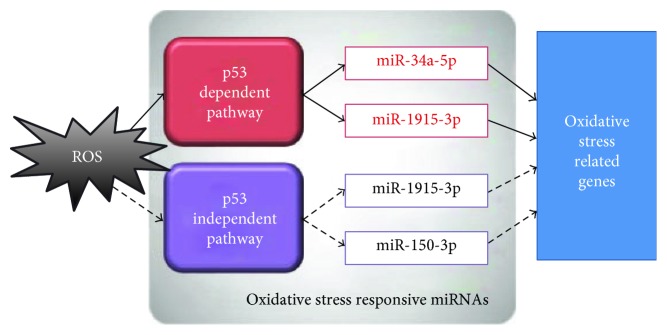
Overview of oxidative stress-responsive miRNAs mechanism in HCC. Oxidative stress-induced miRNA by p53-dependent (miR-34a-5p and miR-1915-3p) or p53-independent (miR-638 and miR-150-3p) pathways. All those oxidative stress-responsive miRNAs inhibit translation of their target genes and involve in oxidative stress process.

For decades, oxidative stress has been reported to impact carcinogenesis. However, the underlying interaction among oxidative stress and carcinogenesis remains unknown. Recently, increasing evidence suggested that some intracellular miRNAs play critical roles in oxidative stress response and carcinogenesis. Therefore, in the current study, we aim to identify several oxidative stress-responsive miRNAs which might involve in liver carcinogenesis. We firstly performed a comprehensive analysis by employing a recently published rank aggregation analysis method [21], to identify oxidative stress-responsive miRNAs. Based on miRNA profiling data, four miRNAs (miR-34a-5p, miR-1915-3p, miR-638, and miR-150-3p) were identified as the common upregulated miRNAs under oxidative stress. Next, we also validated these four oxidative stress-responsive miRNAs in four different HCC cell lines. Specifically, we found that the upregulation of miR-34a-5p and miR-1915-3p, but not miR-638 and miR-150-3p, was depended on p53 status in oxidative stress (Figure 10). Finally, our data also provide clinical evidences for the possible negative regulation of oxidative stress-related genes by these four miRNAs in HCC tissues. We found that the expression levels of those four miRNAs were associated with overall survival time in HCC patients.

miR-34a-5p is one of the most explored miRNAs in carcinogenesis [22]. Although comparatively low levels of miR-34a-5p expression were reported in several cancers [23, 24], discrepancies emerged in HCC. Researchers found that the expression of miR-34a was downregulated during methyl-deficient diet-induced liver carcinogenesis [25]. By contrast, in a chemical-induced HCC model, miR-34a-5p was found to be upregulated [26]. We speculate that miR-34a-5p might be regulated by different mechanisms during liver carcinogenesis. It has been widely accepted that chemotherapeutic agents exhibited anticancer effects by producing ROS in the target tissue. We observed the ROS generation in HCC cells with cisplatin treatment [17]. Bai et al. also found that miR-34a-5p suppressed mitochondrial antioxidative enzymes with a concomitant increase in intracellular ROS level [27]. All above evidences suggested the miR-34-5p expression might be related to ROS level in HCC cells. In the present study, by conducting an oxidative stress model in four HCC cell lines, we confirmed that miR-34a-5p was related to intracellular oxidative stress status. Furthermore, we also found miR-34a-5p was involved in the p53-dependent pathway, which was consistent with previous results [22, 28].

miR-1915-3p was another dysregulated miRNA in various types of cancer, such as prostate cancer, renal cell carcinoma, breast cancer, lung cancer, and colorectal cancer [29–33]. However, the function of miR-1915-3p remains unknown in HCC. The present study revealed for the first time that miR-1915-3p was upregulated by oxidative stress, depending on p53 status in HCC. Recently, a study based on colorectal cancer cells also confirmed our results [32]. The authors [32] reported that miR-1915-3p was involved in response to chemotherapeutic drug-induced DNA damage by targeting *Bcl-2*, the latter of which was an important regulator of oxidative stress [34]. Additionally, they also found that p53 induced the processing of pri-miR-1915-3p to pre-miR-1915-3p, which promote overexpression of mature miR-1915-3p in. These data suggested that, differing from miR-34a-5p, miR-1915-3p expression was regulated by p53 in a transcription-independent way. When focusing on the genes potentially affect oxidative stress, we found that many oxidative stress-related genes might be potential targets of miR-1915-3p. These evidences suggest that by targeting oxidative stress-related genes, miR-1915-3p might possess a significant role in the regulation of oxidative stress response.

miR-638 was previously reported to be associated with cellular senescence [6] and DNA damage [35]. In 2013, Christenson et al. [20] screened the gene expression in miR-638-inhibited fibroblasts and suggested that miR-638 might involve in oxidative stress response. In the present study, we, for the first time, identified miR-638 as an oxidative stress-induced miRNA in HCC cell lines. We found that the elevated expression of miR-638 was not induced by p53. However, the regulation mechanisms of miR-638 during oxidative stress have hardly been investigated. Tay et al. found that miR-638 was encoded by Dnm2 locus and demonstrated that both two components, miR-638 and its host gene Dnm2, were overexpressed in tumorigenesis [36]. Moreover, it was also reported that Dnm2 could be upregulated by radiation-induced oxidative stress [37]. Therefore, we hypothesized that oxidative stress might promote transcription of host gene Dnm2 and miR-638 was cooperatively upregulated. Furthermore, our data also showed that HCC patients with high risk had a higher expression level of miR-638 than low risk group. However, the association between miR-638 and cancer prognosis was inconsistent. Some studies showed that the downregulation of miR-638 was involved in malignant phenotypes of osteosarcoma, gastric carcinoma, and colorectal carcinoma [38–40]. By contrast, Bhattacharya et al. [41] reported that miR-638 promoted melanoma progression. Besides, Ren et al. [42] also found that miR-638 acts as an oncogene and promotes cell proliferation and metastasis in esophageal squamous cell carcinoma and breast cancer cells. Considering our data did not get a statistically significant *P* value, the jury must refrain from drawing a firm conclusion until a well-designed clinical study confirms or refutes the above findings.

Additionally, we also observed that miR-150-3p was induced by oxidative stress and was not depended on p53 overexpression. However, its functions in oxidative response and liver carcinogenesis have yet to be unravelled. It was reported that miR-150-3p could be induced by NF-kB activation [43]. Considering the important roles of NF-kB pathway in oxidative stress response and inflammation [44], investigating the underlying interaction between NF-kB and miR-150-3p will be of particular interest to study the molecular mechanisms of oxidative stress response in liver carcinogenesis.

Taken together, we identified four oxidative stress-responsive miRNAs (miR-34a-5p and miR-1915-3p, but not miR-638 and miR-150-3p) and further demonstrated p53-dependent and p53-independent mechanisms of miRNA expression regulation in HCC. Given a critical role for oxidative stress in carcinogenesis, our present findings have significant clinical implications. These miRNAs might offer new potential strategy for cancer diagnosis and prognosis.

## Supplementary Material

Supplementary Table. Table S1 Characteristics of studies included for meta-analysis of hydrogen peroxide-responsive miRNAs.

## Figures and Tables

**Table 1:** The signaling pathway enrichments of validated targets of four miRNAs.

KEGG signaling pathways	*P* value
KEGG_04115: p53 signaling pathway	2.24*E* − 10
KEGG_04310: Wnt signaling pathway	4.83*E* − 09
KEGG_04330: notch signaling pathway	1.34*E* − 06
KEGG_04630: Jak-STAT signaling pathway	3.83*E* − 04
KEGG_04722: neurotrophin signaling pathway	1.73*E* − 03
KEGG_04340: hedgehog signaling pathway	1.89*E* − 03
KEGG_04010: MAPK signaling pathway	3.33*E* − 03
KEGG_04370: VEGF signaling pathway	4.06*E* − 03
KEGG_04350: TGF-beta signaling pathway	4.98*E* − 03
KEGG_04660: T cell receptor signaling pathway	9.59*E* − 03
KEGG_04150: mTOR signaling pathway	2.14*E* − 02
KEGG_04920: adipocytokine signaling pathway	3.27*E* − 02
KEGG_03320: PPAR signaling pathway	3.36*E* − 02
