# Supplementary material for: Identification of Four Oxidative Stress-Responsive MicroRNAs, miR-34a-5p, miR-1915-3p, miR-638, and miR-150-3p, in Hepatocellular Carcinoma
Source: Oxid Med Cell Longev. 2017 Jul 24;2017:5189138. doi: 10.1155/2017/5189138 (PMC5546075; doi:10.1155/2017/5189138)
Supplement: Supplementary file 1 — Supplementary Table. Table S1 Characteristics of studies included for meta-analysis of hydrogen peroxide-responsive miRNAs. [file 5189138.f1.pdf]

Supplementary Tables

Table S1 Characteristics of studies included for meta-analysis of hydrogen peroxide-responsive miRNAs

Author (year)	Species	Cell type	H_2O_2 treatment		Ref.
			Concentration	Time	
Lin (2009)	Mouse	VSMCs	200μM	6h	[5]
Maes (2009)	Human	WI-38 fibroblasts	200μM	NA	[6]
Magenta (2011)a	Human	HUVECs	200μM	8h	[7]
Magenta (2011)b	Human	HUVECs	200μM	24h	[7]
Mateescu (2011)	Mouse	Fibroblasts	400μM	24h	[8]
Xu (2012)	Mouse	Neuron	200μM	6h	[9]
Howell (2013)	Human	ARPE-19	200μM	18h	[10]
Jong (2013)	Human	HUVECs	200μM	2hx2d	[11]
Kim (2014)	Human	Human dermal papilla cells	750μM	24h	[12]
Cross (2015)	Human	CRL-1584	25μM	4h	[13]
Luo (2016)	Human	HepG2	600μM	24h	[14]

VSMC: vascular smooth muscle cells; HUVEC: human umbilical vein endothelial cells; NA, not available.
